# Schwann Cells in Neuromuscular Disorders: A Spotlight on Amyotrophic Lateral Sclerosis

**DOI:** 10.3390/cells14010047

**Published:** 2025-01-03

**Authors:** Kathryn R. Moss, Smita Saxena

**Affiliations:** 1 Department of Physical Medicine and Rehabilitation, University of Missouri School of Medicine, Columbia, MO 65211, USA; 2 NextGen Precision Health, University of Missouri, Columbia, MO 65211, USA

**Keywords:** ALS, CMT1, Schwann cell, myelin, terminal Schwann cells, NMJ, satellite glial cells

## Abstract

Amyotrophic Lateral Sclerosis (ALS) is a complex neurodegenerative disease primarily affecting motor neurons, leading to progressive muscle atrophy and paralysis. This review explores the role of Schwann cells in ALS pathogenesis, highlighting their influence on disease progression through mechanisms involving demyelination, neuroinflammation, and impaired synaptic function. While Schwann cells have been traditionally viewed as peripheral supportive cells, especially in motor neuron disease, recent evidence indicates that they play a significant role in ALS by impacting motor neuron survival and plasticity, influencing inflammatory responses, and altering myelination processes. Furthermore, advancements in understanding Schwann cell pathology in ALS combined with lessons learned from studying Charcot–Marie–Tooth disease Type 1 (CMT1) suggest potential therapeutic strategies targeting these cells may support nerve repair and slow disease progression. Overall, this review aims to provide comprehensive insights into Schwann cell classification, physiology, and function, underscoring the critical pathological contributions of Schwann cells in ALS and suggests new avenues for targeted therapeutic interventions aimed at modulating Schwann cell function in ALS.

## 1. Introduction

The peripheral nervous system (PNS) contains a diverse repertoire of glial cells of which Schwann cells are the best characterized [1]. Schwann cells are typically classified as myelinating Schwann cells, non-myelinating Schwann cells, or satellite glia. Non-myelinating Schwann cells are further categorized as Remak Schwann cells or terminal Schwann cells. Schwann cells play critical roles in the PNS, and their dysfunction has dire effects. In this review, we briefly summarize the development and maintenance of each type of Schwann cell and discuss their role in neurodegenerative disease including Amyotrophic Lateral Sclerosis (ALS).

### 1.1. Myelinating Schwann Cells

**Figure 1:**
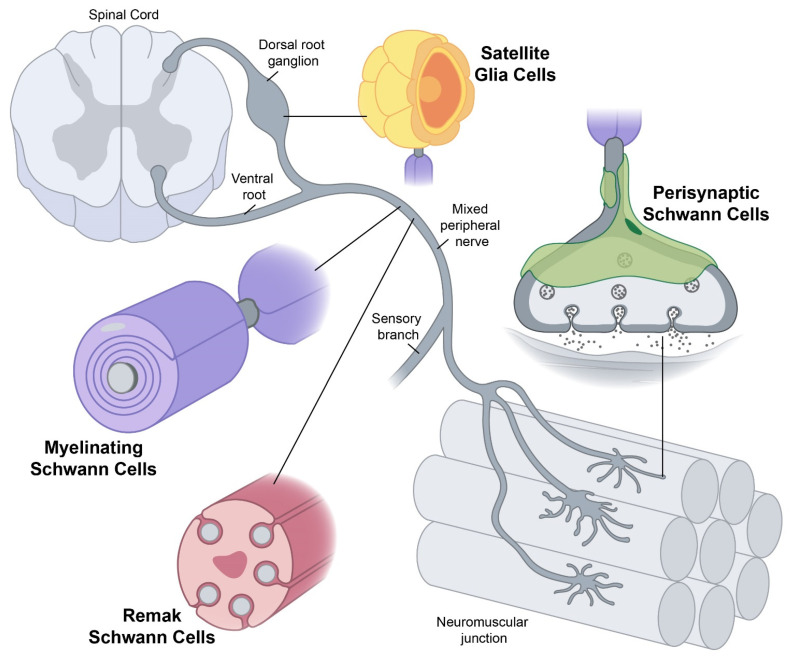
Multiple types of Schwann cells in the peripheral nervous system. The location and morphology of Schwann cells, including myelinating Schwann cells, Remak Schwann cells (adapted with permission from Ref. [10] 2005, Elsevier Inc.), perisynaptic Schwann cells, and satellite glia (adapted with permission from Ref. [17]) are depicted.

Myelinating Schwann cells spiral around medium to large caliber (>1 μm) peripheral nerve axons to form a compact multilamellar myelin sheath that covers one segment of a single axon and enabling saltatory conduction of action potentials (Figure 1). Schwann cell myelination occurs on A and B fibers which includes mostly motor neuron axons but also some large sensory neuron axons and medium autonomic neuron axons [2]. The developmental lineage of myelinating Schwann cells is as follows: neural crest → Schwann cell precursor → immature Schwann cell → pro-myelinating Schwann cell → myelinating Schwann cell [3]. The transition from neural crest to Schwann cell precursor is driven by dramatic gene expression changes which includes hundreds of genes (i.e., the upregulation of *Myelin Protein Zero* [*MPZ*], *Peripheral Myelin Protein 22* [*PMP22*], *Proteolipid Protein* [*PLP*], *Growth Associated Protein 43* [*GAP43*], etc.) [3,4]. Schwann cell precursors migrate along growing peripheral nerve axons and they are dependent on signals from these nascent axons for survival (primarily Neuregulin 1 type III [NRG1-III]) [1,3]. Schwann cell precursors are remarkably multipotent with the ability to differentiate into several cell types [1,3]. The differentiation of Schwann cell precursors to immature Schwann cells again involves gene expression changes (i.e., the upregulation of *S100* and *Glial Fibrillary Acidic Protein* [*GFAP*], and the downregulation of *Transcription Factor AP2* [*TFAP2A*] and *N-Cadherin*) [4,5]. Immature Schwann cells no longer require axonal signals for survival, develop a basal lamina (a thin layer of extracellular matrix), have elongated morphologies, and demonstrate reduced migration but increased proliferation [3]. Immature Schwann cells carry out the remarkable process of radial sorting in which axons are classified by their caliber to initiate the programs for generating myelinating and Remak Schwann cells. This stepwise process begins by immature Schwann cells forming groups (3–8 cells) which organize a shared basal lamina and bundle several axons [6]. Immature Schwann cells then extend lamellipodia-like processes between the bundled axons to select and segregate larger caliber axons to the exterior edges of the bundle. Immature Schwann cells proliferate and continue to subdivide the axon bundle until medium to large caliber axons acquire a 1:1 relationship with a pro-myelinating Schwann cell. Pro-myelinating Schwann cells establish their own basal lamina (called defasciculation) and go on to form myelinating Schwann cells. When radial sorting is complete, the small axons (<1 μm diameter) remaining in the bundle will be engulfed by Remak Schwann cells to form Remak bundles (discussed in Section 1.2.1). Given the complex morphogenetic processes required for radial sorting and myelination, it is not surprising that intricate signaling pathways and extensive gene expression changes govern them (see several excellent reviews which also contain signaling pathway schematics [6,7,8,9]). These include interactions between Schwann cells and the basal lamina as well as interactions between Schwann cells and axons that work in concert to activate signaling cascades and upregulate promyelinating transcription factors like Sox10 and Krox20. Axonal NRG1-III is a key regulator of myelin sheath thickness [10] and mechanical forces acting on the HIPPO pathway control myelin sheath internodal length [11,12].

The spiraling and compaction of myelinated Schwann cells continues through the coordination of several cellular processes including plasma membrane expansion, proper stoichiometric insertion of compact myelin proteins and lipids, and F-actin assembly and disassembly [8,13]. The resulting myelin sheaths demonstrate both longitudinal (axial) and radial polarity. These sheaths are not continuous compact multilamellar structures but instead contain discrete domains including nodes, paranodes, and juxtaparanodes at the Node of Ranvier and irregularly spaced Schmidt–Lanterman Incisure transport channels along the length of the internode [8]. There are also distinct domains at the interior or inner-most wrap of the myelin sheath (adaxonal membrane, inner mesaxon, and inner collars) and the exterior or outer-most wrap (abaxonal membrane, outer mesaxon, outer collars, Cajal bands, appositions, and the nucleus) [8]. There has been progress in understanding the processes that orchestrate the complex architecture of peripheral nerve myelin, and the list of resident proteins localized to the different myelin domains continues to grow but there remains much that is unknown [14,15]. Additionally, disentangling the contributions of myelin signaling pathways and gene expression to peripheral nerve myelin development as compared to maintenance can be difficult, but there appear to be some factors that are only required for development but not maintenance (i.e., ErbB2) [16]. Furthermore, some of the most critical insights regarding myelinating Schwann cells have come from nature because several types of inherited neuropathy are caused by mutations affecting these cells (discussed in Section 2.1).

### 1.2. Non-Myelinating Schwann Cells

Non-myelinating Schwann cells include Remak Schwann cells and terminal Schwann cells, and these cells clearly play critical roles in the PNS even though they are less characterized as compared to their myelinating counterparts.

#### 1.2.1. Non-Myelinating Remak Schwann Cells

Remak Schwann cells engulf small caliber (<1 μm) peripheral nerve axons to provide them with trophic and metabolic support (Figure 1) [18]. These Remak bundles contain C fibers which include sensory neuron and autonomic neuron axons [2]. Remak Schwann cells are developed from the same lineage as myelinating Schwann cells but arise from an alternative fate at the immature Schwann cell stage [1,18]. As mentioned previously, small axons (<1 μm diameter) remaining after radial sorting will be engulfed by Remak Schwann cells to form Remak bundles. However, the fate decision between pro-myelinating and Remak Schwann cells remains unclear. Axonal NRG1-III clearly plays an important role in Remak bundle development [19] but NRG1-III signaling does not appear to be the sole signaling pathway required for fate specification [18]. Additionally, myelinating Schwann cells and Remak Schwann cells demonstrate distinct gene expression profiles which include changes in cell adhesion molecules, receptors, and transcription factors [8]. A hallmark of mature Remak bundles is the complete ensheathment of each axon in the bundle such that axons are separated from each other by Remak Schwann cell membrane [18]. Remak Schwann cell maturation and maintenance also demonstrates similarities and differences to that of myelinating Schwann cells. Similarities include PI-3Kinase/AKT-1 and Gpr126 signaling, and a difference includes the expression of Neuropathy Target Esterase (NTE) [18,20].

#### 1.2.2. Non-Myelinating Terminal Schwann Cells

Terminal Schwann cells are present at all the innervation targets of peripheral nerve axons including neuromuscular junctions in skeletal muscle and various sensory end-organ structures in skin (i.e., Meissner corpuscles, Pacinian corpuscles, hair follicles, and free nerve endings) [21]. Terminal Schwann cells are believed to be derived from neural crest through a Schwann cell precursor lineage like myelinating Schwann cells and Remak Schwann cells but factors driving terminal Schwann cell fate specification remain unclear [21,22,23]. However, the development and maturation of terminal Schwann cells parallels the maturation of the end-organ structure. We will solely focus on neuromuscular junction (NMJ) terminal Schwann cells, called perisynaptic Schwann cells, for the remainder of this review due to their relevance to ALS. NMJs are chemical synapses between myelinated motor neuron axons and skeletal muscle fibers, and they are comprised of presynaptic axon terminal boutons, synaptic clefts, and postsynaptic muscle end plates (Figure 1) (as referenced in these excellent reviews [24,25]). Terminal boutons contain active zones filled with Ca2+ channels, synaptic vesicles loaded with acetylcholine (ACh), and proteins that facilitate synaptic vesicle docking and fusion. The space between a terminal bouton and the muscle end plate is called the synaptic cleft which contains acetylcholinesterase to catabolize excess ACh that is released in response to action potentials. Muscle end plates are made up of junctional folds with apical clustered nicotinic ACh receptors that open upon binding ACh and trigger the transmission of the action potential from the axon to the muscle. Each adult mouse NMJ contains approximately three perisynaptic Schwann cells that envelop the axon terminal arbor and although they are not required for NMJ innervation, they do support efficient NMJ innervation, maturation, and synaptic elimination and they serve as an insulator to separate the NMJ from surrounding tissues [21,26]. The gene expression profiles of perisynaptic Schwann cells revealed an enrichment of genes involved in cell adhesion, phagocytosis, and extracellular matrix formation [27]. Perisynaptic Schwann cells are also required for proper NMJ transmission which involves detecting synaptic activity and modulating synaptic plasticity but the mechanisms for these processes are not well defined [21,26]. In addition, perisynaptic Schwann cells play important roles during reinnervation following nerve injury by serving as tracts for regenerating axons to extend along to promote reinnervation of original synaptic sites [28,29]. Perisynaptic Schwann cells are the best characterized Schwann cell type in terms of their involvement in ALS pathogenesis (discussed in Section 2.2.2).

### 1.3. Satellite Glial Cells

Satellite glia are flattened cells that tightly wrap around neuronal cell bodies in sensory and autonomic ganglia (Figure 1). Dorsal root ganglia (DRG) contain the cell bodies for all sensory neurons that innervate targets below the neck whereas the trigeminal and nodose ganglia contain sensory neurons that innervate the head [30]. The number of satellite glial cells per neuron is positively correlated with the size of the soma with estimates of 4–12 satellite glia per DRG neuron reported in mice [31]. Satellite glial cells are derived from neural crest through a Schwann cell precursor lineage similar to myelinating and non-myelinating Schwann cells, but they are believed to developmentally stall or fate switch due to their interactions with DRG neuronal soma [32]. Although the signaling pathways and gene expression changes that drive satellite glial cell fate specification remain unclear, omics studies reveal that these cells express markers similar to Schwann cell precursors and their CNS counterpart, astrocytes [1,31]. Another shared feature between satellite glia and astrocytes is their ability to be activated in response to injury and inflammation. The satellite glia-neuronal soma unit is enclosed by a single basal lamina and the satellite glia in one unit are connected to one another through gap junctions enabling them to control neuronal homeostasis, connectivity, and synaptic transmission [1,31,33,34]. Upon injury and inflammation, satellite glia become activated which includes the upregulation of Glial Fibrillary Acidic Protein (GFAP) and increased gap junction connectivity. Activated satellite glia release pro-inflammatory cytokines which leads to increased excitability and firing of the encircled neuron contributing to neuropathic pain [1,31,33,34]. Like fate specification, not much is known about the maturation and maintenance of satellite glia.

## 2. Schwann Cell Dysfunction in Disease

Schwann cells have diverse and important functions in the PNS. In addition to serving as insulators to promote saltatory conduction, myelinating Schwann cells also organize the node of Ranvier and the axonal cytoskeleton, provide axons with trophic and metabolic support, and protect axons from insults [35]. The primary role for Remak Schwann cells is to provide axons with trophic and metabolic support but they also function as immunocompetent cells [36]. Terminal Schwann cells are generally involved in the development, maintenance, and plasticity of end-organ structures and, as described above, perisynaptic Schwann cells at NMJs are also involved in synaptic transmission [21,26]. Satellite glial cells exert several effects on their encircled neuronal soma including metabolic support, protection from insults, and the control of synaptic transmission [30]. Therefore, it is not surprising that human diseases caused by the dysfunction of myelinating Schwann cells [37], terminal Schwann cells [38], and satellite glial cells [33] have been identified and result in dramatic PNS functional impairment that negatively impacts a patient’s quality of life. We will first explore Charcot–Marie–Tooth disease Type 1, one of the most common inherited disorders affecting Schwann cells, to provide a foundation for understanding the role of these cells in ALS pathogenesis and the potential therapeutic benefits of targeting them in ALS treatment.

### 2.1. Charcot–Marie–Tooth Disease Type 1

Charcot–Marie–Tooth disease (CMT) is the most common inherited peripheral neuropathy affecting approximately 1:2500 people and causing length-dependent sensorimotor defects including muscle weakness and loss, foot deformities, abnormal sensation, and balance deficits [37]. CMT Type 1 (CMT1) is diagnosed by slow nerve conduction velocity (<35 m/s) and is caused by dominant and recessive as well as autosomal and X-linked mutations of genes affecting myelinating Schwann cells. There are currently 10 CMT1 genes identified and CMT1 accounts for approximately 83% of genetically defined CMT cases including CMT1X and Hereditary Neuropathy with Liability to Pressure Palsies (HNPP) [37,39,40]. These genes and their corresponding CMT subtypes are listed in Table 1 along with information about the gene function, mutation location, and disease severity [37]. *Peripheral myelin protein 22* (*PMP22*), *myelin protein zero* (*MPZ*/*P0*), and *gap junction protein beta 1* (*GJB1*/*Cx32*) are by far the most common CMT genes and thus will be discussed in additional detail.

The *PMP22* gene is the most common CMT gene accounting for approximately 65% of genetically defined CMT cases and causes three CMT subtypes: CMT1A (49%), HNPP (15%, although likely an underestimate), and CMT1E (1%) [39,40]. CMT1A is generally considered to present with classic CMT symptoms whereas HNPP is milder and CMT1E can present as mild, classic, or severe CMT [41]. The *PMP22* gene is upregulated in Schwann cell precursors by multiple transcription factors including Sox10 and Krox20 [4,41] and is proposed to be upregulated further as myelinating Schwann cells develop and contact axons. PMP22 protein also undergoes complex post-translational modification, and it is estimated that most newly synthesized PMP22 is degraded (~70%) to create a stringent checkpoint for proper PMP22 processing [41,42]. However, most of these studies were performed in cultured cells so it is difficult to interpret how this relates to a myelin sheath with a highly polarized plasma membrane. The physiological function of PMP22 protein remains unclear but current evidence suggests that it plays an undefined role in cell adhesion [43,44,45,46,47,48]. With this background in mind, several non-exclusive pathomechanisms have been proposed for CMT1A with some variations of these considered for HNPP and CMT1E including altered myelin architecture and function due to abnormal PMP22 membrane stoichiometry, PMP22 protein aggregation/ER stress, disrupted axonal architecture and transport, Schwann cell death, and secondary axon degeneration due to myelin dysfunction [37,41].

The *GJB1* gene is the second most common CMT gene accounting for approximately 14% of genetically defined CMT cases and causes CMT1X [39,40]. CMT1X is generally considered to present with classic CMT symptoms but with males more severely affected than females due to an X-linked inheritance [37,49]. Additionally, CMT1X patients generally experience higher rates of hand deformity as compared to classic CMT and frequently exhibit CNS symptoms [50]. The *GJB1* gene encodes Connexin32 protein (Cx32) which is upregulated late in the Schwann cell developmental lineage, potentially only at the myelinating Schwann cell stage [51,52,53]. The transcription factors Sox10 and Krox20 also promote *GJB1* transcription [54]. Cx32 is a gap junction protein that forms hemichannels in areas of non-compact myelin-like paranodes and Schmidt–Lanterman Incisures [55]. Cx32 is suggested to facilitate the transport of metabolites and signaling molecules throughout the myelin sheath and disrupting this process is a proposed pathomechanism for CMT1X [55].

The *MPZ* gene is the third most common CMT gene accounting for approximately 7% of genetically defined CMT cases and causes three CMT subtypes: CMT1B (4%) and CMT2I/J (3%) [39,40]. CMT1B can present as mild, classic, or severe CMT whereas CMT2I/J is more mild given that it typically has an adult-onset, but it can progress rapidly [37,56]. Of note, CMT2I and CMT2J are often grouped together because they are diagnosed as CMT2 by nerve conduction studies (mild myelin defect but dramatic axonal deficits) but CMT2J has the distinguishing features of hearing loss and pupillary abnormalities [37,56]. The *MPZ* gene is expressed early in the Schwann cell developmental lineage given that it is detected in a subpopulation of neural crest cells [3]. In addition, like *PMP22*, *MPZ* expression is upregulated in Schwann cell precursors by the multiple transcription factors including Sox10 and Krox20 and is proposed to be upregulated further as myelinating Schwann cells develop [3,57]. MPZ protein (also called P0) is only expressed in the myelinating Schwann cell developmental lineage, and it is the most enriched protein in compact myelin [37,56]. It serves as an adhesion protein that is critical for the compaction of myelin lamellae [37,56]. Three distinct pathomechansims are proposed to account for the three different clinical presentations of *MPZ*-mediated CMT [37,56]. MPZ mutations causing classic CMT are proposed to result in a loss of function affecting the adhesive function of MPZ in compact myelin. MPZ mutations causing severe CMT are proposed to result in a toxic gain of function which can cause ER stress and activate the unfolded protein response. Furthermore, pathomechanisms of MPZ mutations causing mild/adult-onset CMT remain unclear, but it seems logical that myelin sheath functions that are independent from the canonical insulation function may be disrupted (i.e., axonal homeostasis including trophic and metabolic support and axonal architecture regulation).

The clinical and scientific exploration of CMT1 has revealed important insights into the role of several genes in the development and maintenance of myelinating Schwann cells. Given that a diagnosis of CMT1 is based on myelinating Schwann cell dysfunction (slow nerve conduction velocity), it is not surprising that the vast majority of research on this disease has been focused on myelinating Schwann cells. However, it is intriguing to consider how Remak Schwann cells, terminal Schwann cells, and satellite glial cells may be affected in CMT1 given the shared developmental lineage of these cells.

### 2.2. Amyotrophic Lateral Sclerosis

Amyotrophic Lateral Sclerosis (ALS) is an adult-onset neurodegenerative disease defined by the gradual degeneration of upper and lower motor neurons leading to spasticity, hyperreflexia, fasciculation, and eventual muscle atrophy due to the denervation of motor axons at NMJs [58,59,60]. Frontotemporal Dementia (FTD) is yet another neurodegenerative disease that mainly damages the frontal and temporal lobes of the brain, resulting in progressive changes in behavior, personality, and speech [58,61,62]. Based on overlapping clinical, genetic, and epidemiological data, ALS and FTD have been classified as the two ends of the same disease spectrum [63,64,65,66]. Approximately 15% of FTD patients exhibit symptoms of classical ALS disease involving motor symptoms, whereas up to 50% of ALS patients have symptoms of FTD associated with behavioral and personality changes [67]. More than 50 potential causal or disease-modifying genes have been linked to familial forms of ALS (fALS). However, pathogenic variants in *SOD1*, *C9ORF72*, *FUS*, and *TARDBP* genes occur most commonly, while disease-causing variants in other genes are relatively rare [68,69]. In 90% of ALS cases, the etiology remains unexplained and is termed sporadic ALS (sALS); however, genetic risk factors are thought to contribute to the risk of developing sALS, with heritability accounting for 60% of the cases, these estimates obtained from studies done on twins [70,71].

SOD1 mutations and disease mechanism:

The *SOD1* gene encodes Cu/Zn superoxide dismutase 1 and more than 100 point mutations in this gene have been identified which induce conformational and functional changes, leading predominantly to a toxic gain of function (GOF) of the mutant protein involving several pathological mechanisms. These include oxidative stress via the upregulation of reactive oxygen species, endoplasmic reticulum stress, excitotoxicity, and mitochondrial dysfunction [72,73,74,75,76]. However, it remains unclear whether the soluble or aggregated forms of SOD1 are responsible for exerting toxicity. Moreover, non-native formations of wild-type SOD1 have been observed in small granular SOD1-immunoreactive inclusions in sALS patient motor neurons and in patients harboring the *C9ORF72* repeat expansion and pathogenic variants in other ALS-associated genes [77,78], suggesting that both mutant and wild-type SOD1 forms harbor the potential to misfold and cause ALS.

TDP-43 mutations and disease mechanisms:

In yet another genetic mutation of ALS, TDP-43 (TAR DNA-binding protein 43) is commonly found in pathological aggregates in ALS and FTD due to the cytoplasmic accumulation of TDP-43 together with a loss of nuclear TDP-43, thus leading to the proposed disease mechanism involving a loss of normal TDP-43 function in the nucleus, a toxic GOF, or both. The maintenance of TDP-43 homeostasis is crucial for normal cellular function. The nuclear depletion of TDP-43 leads to the upregulation of TDP-43 [79], whereas excess TDP-43 in the cytoplasm causes the accumulation of inclusion bodies leading to cellular dysfunction. Moreover, nuclear depletion causes the widespread dysregulation of mRNA metabolism, with TDP-43 knockdown shown to lead to the differential splicing or expression of hundreds of targets [80,81,82]. In addition to abnormal distribution and aggregation of TDP-43 in ALS, several post-translational modifications (PTMs) are linked to pathologic TDP-43, including proteolytic cleavage, ubiquitination, and phosphorylation [83,84].

FUS mutations and disease mechanisms:

Around 50 autosomal dominant FUS variants have been identified in ALS patients, the majority of mutations are missense mutations with some rare insertions, deletions, splicing, and nonsense mutations [85]. The pathologic cytoplasmic redistribution of nuclear FUS leads to a loss of its normal function in the nucleus [86]. Additionally, both cytoplasmic aggregates as well as the accumulation of the soluble FUS in the cytoplasm mediate cellular toxicity [70,87,88]. Pathogenic cytoplasmic FUS distribution alters stress granule dynamics, causes splicing defects and DNA damage, and compromises FUS autoregulation [70,89,90,91,92,93].

C9ORF72 mutation and disease mechanisms:

In 2011, a hexanucleotide repeat expansion in the non-coding region of the *C9ORF72* gene was identified as a disease mutation common between ALS and FTD. This GGGGCC (G_4_C_2_) hexanucleotide repeat expansion is located in the first intron in the reading frame 72 of chromosome 9 (*C9ORF72*) in the non-coding region between exons 1 and 1b [94,95]. Healthy individuals carry less than 30 G_4_C_2_ repeats, while ALS/FTD patients with *C9ORF72* pathogenic mutations carry 400 to a few thousand G_4_C_2_ repeats [96]. An assessment of the postmortem tissue of C9ORF72 ALS/FTD patients revealed a significant decrease in the total C9ORF72 transcript as well as protein levels compared to healthy controls [95,97,98,99,100]. A reduction of C9ORF72 levels enhances neurodegeneration caused by the gain of toxicity of the repeat expansion [101] and dipeptide repeat proteins (DPRs) [102]. The second pathogenic mechanism involves a GOF effect resulting from the formation of toxic RNA foci derived from repeat expansion transcripts (reviewed in [103]). These RNA foci are a defining pathological feature of C9ORF72-associated ALS/FTD. Both sense and antisense RNA foci have been detected in various regions of the central nervous system in C9ORF72 ALS/FTD patients and across multiple disease models [95,101,104,105]. Studies have shown that RNA foci can sequester essential RNA-binding proteins (RBPs), potentially impairing their localization and function [101,106,107]. Furthermore, RNA foci have been associated with TDP-43 mislocalization in both patients and mouse models of C9ORF72 ALS/FTD [108]. The third mechanism involves a gain-of-function (GOF) resulting from the formation and accumulation of dipeptide repeat proteins (DPRs) through the repeat-associated non-AUG (RAN) translation of hexanucleotide repeat sequences from both sense and antisense strands (reviewed in [109,110]). The contributions of both loss-of-function (LOF) and GOF mechanisms to C9ORF72 ALS/FTD pathophysiology have been extensively studied across various model systems. While multiple mechanisms may collectively drive the pathology of C9ORF72 ALS/FTD, the presence of DPRs in neurons strongly suggests that they play a critical role in disease progression. Disruptions in autophagy-lysosomal pathways, dysfunctional nucleocytoplasmic transport, RNA toxicity, and toxicity from aggregation-prone DPRs, which sequester vital proteins, thus cause a homeostatic imbalance [99,110,111].

Notably, ALS onset is usually focal, eventually involving both upper or lower limbs coupled together with bulbar or respiratory regions. The resulting disease progression affects adjacent body regions, resulting in global muscle weakness, with respiratory dysfunction representing the terminal phase of the disease [112,113]. Understanding the relationship between upper and lower motor neuron dysfunction is critical for unraveling ALS pathogenesis, and three opposing theories have been proposed [114,115]. Firstly, it has been suggested that ALS originates at a cortical level, with corticomotoneuronal hyperexcitability spreading neuronal degeneration via a transsynaptic anterograde mechanism [116,117]. Yet another theory stipulates that lower motor neuron dysfunction is the primary event that afflicts the motor neuron synapse at the NMJ, thereby inducing a retrograde dying back process termed distal axonopathy [118,119]. Lastly, the random and parallel degeneration of the upper and lower motor neuron leads to ALS symptoms within defined and established disease-affected anatomical regions [120]. Notably, much effort has been expended into motor axon degeneration at the NMJ. Using the mouse models of ALS mutations (G93A SOD1 and G85R SOD1), it was established that ALS disease encompasses selective motor axon vulnerability patterns, defined by predictable episodes of the sudden pruning of physiological axon subtypes in the target area and compensation via the sprouting of resistant motor axons [121]. Notably, these studies highlighted that motor neuron synapses at the NMJ differ markedly in their physiological plasticity capacity. Motor axon terminals innervating slow muscle fibers harbor immense regeneration capacity, thus sprouting vigorously following synaptic loss or the denervation of target muscle fibers. In contrast, those innervating fast-fatigable muscle fibers largely lack sprouting capabilities [121,122,123]. Moreover, it has largely been recognized that ALS mutations impact the sprouting capacity of motor axons both in rodent models as well as in human ALS patients [121,122,123,124,125,126,127].

The genetic mutations identified in fALS families were used to develop several ALS models. Rodent models are crucial, as mice and rats harboring human disease mutations permit the study of a disease in a living mammal, thus enabling a near-faithful recapitulation of the human condition. In 1993, Rosen et al. identified mutations in the *SOD1* gene in familial ALS (fALS) cases, establishing *SOD1* as the first gene linked to ALS [128]. Following this breakthrough, research efforts focused on understanding the role of *SOD1* in ALS pathogenesis, leading to the development of the first genetic ALS model in 1994. Gurney et al. created a transgenic mouse model overexpressing the ALS-associated *SOD1-G93A* mutation [129,130]. These mice exhibited hallmark ALS characteristics, including motor neuron loss, paralysis, and early mortality. This model remains one of the most widely used tools in ALS research. Over time, additional *SOD1* mouse models were developed, incorporating mutations such as *SOD1-G37R*, *SOD1-G85R*, *SOD1-G86R*, *SOD1-D90A*, *SOD1-H46R*, and *SOD1-D83G* [131,132,133,134,135,136]. These models display a range of ALS-like phenotypes, often influenced by the level of mutant *SOD1* expression. Like SOD1–linked ALS mutations, mouse models overexpressing mutant forms of TDP-43 (such as TDP-43-A315T or TDP-43-M337V) have been developed to mimic the pathological features of ALS. These models exhibit motor neuron degeneration, muscle atrophy, and, crucially, TDP-43 proteinopathy akin to what is observed in human ALS [137]. In rat models of FUS, progressive motor impairments, respiratory dysfunction [138], paralysis, axonal degeneration, neuronal loss in the cortex and hippocampus, protein aggregation, and glial activation are observed [87]. In mice, studies report protein aggregation in motor neurons leading to neurodegeneration [139], damage to neuromuscular junctions [140], and disrupted protein transport between the endoplasmic reticulum and Golgi complex in neuronal cells [141]. Mouse models expressing the C9ORF72 mutation exhibit neuromuscular junction damage, hippocampal dispersion, apoptosis, gait deficits, and cognitive impairments [142,143]. Additional findings include dipeptide and TDP-43 protein inclusions, a loss of Purkinje and cortical neurons, astrogliosis, weight loss, and behavioral changes such as hyperactivity, anxiety, and motor deficits [108,142,144,145]. In contrast, transgenic C9ORF72 knockout mice do not show evidence of neurodegeneration or motor impairments [145]. These mouse models have also provided valid information about the non-cell autonomous nature of ALS disease onset and progression mainly on the contribution of nonneuronal cells in the pathophysiology of ALS. Considering the conserved deficits at the NMJ, along with axonal deficits and motor neuron axon loss in ALS, we elaborate on the involvement of the Schwann cells in ALS in this review.

#### 2.2.1. Myelinating Schwann Cells and Amyotrophic Lateral Sclerosis

Myelinating Schwann cell disruption is becoming appreciated as a significant aspect of ALS pathology. Motor neuron degeneration in ALS can lead to secondary effects on myelinating Schwann cells on peripheral axons. Studies have shown that demyelination and remyelination disturbances are present in ALS patients, suggesting that Schwann cells may become dysfunctional because of the disease [146]. Autopsy studies of ALS patients have revealed areas of demyelination in peripheral nerves [147,148]. These areas correspond to regions where motor neurons have degenerated, indicating that the loss of neuronal input leads to the deterioration of nearby myelin sheathes. The observed demyelination in ALS patients suggests that the propagation of electrical signals is disrupted, leading to slower and less efficient nerve conduction, thus contributing to the muscle weakness and atrophy characteristic of ALS, as motor neurons are no longer able to effectively communicate with muscle fibers. Additionally, the loss of myelin not only impairs nerve function but can also make axons more vulnerable to degeneration. Without adequate myelination, axons may undergo further damage, accelerating the progression of ALS. Myelinating Schwann cells are known to respond to nerve injury by promoting repair and remyelination. However, in ALS, this repair response may be impaired or insufficient [149]. Research indicates that while myelinating Schwann cells attempt to remyelinate damaged axons, the progressive nature of motor neuron degeneration in ALS overwhelms this regenerative capacity, leading to further nerve dysfunction [150]. Furthermore, research has shown that myelinating Schwann cells in the SOD1-G93A and TDP-43 model are additionally prone to apoptosis [69]. This loss of myelinating Schwann cells reduces the overall support available to motor neuron axons, contributing to their degeneration. The stress induced by mutant SOD1 in Schwann cells increases oxidative stress and mitochondrial dysfunction, leading to cell death. Even before dying, myelinating Schwann cells in the SOD1-G93A model show dysfunctional behavior, including altered gene expression, reduced metabolic support to axons, and the inability to efficiently clear debris from degenerating axons [151,152]. This dysfunction further weakens the motor neuron-Schwann cell unit and accelerates neurodegeneration. In the most commonly occurring genetic mutation responsible for 50% of familial forms of ALS-FTD, C9ORF72, a significant myelin and lipid loss in frontal white matter was observed due to defective myelin lipid catabolism and impaired oligodendrocytes. While no investigation of myelinating Schwann cells was performed, it is likely that a myelin deficit would also impact either the myelination capacity or repair responses of Schwann cells on peripheral motor axons [153].

Studies have shown that early demyelination events occur before significant motor neuron loss is evident in the SOD1-G93A mice and ALS patients [154]. This early demyelination is often observed in peripheral nerves and is associated with Schwann cell dysfunction. Further, even before the onset of clinical symptoms, abnormalities in the axonal structure and myelin integrity can be detected. These changes suggest that Schwann cells are affected early in the disease process, possibly due to the toxic effects of the mutant SOD1 protein. Moreover, as the disease progresses, more extensive demyelination occurs, particularly in motor nerves. This demyelination is characterized by the thinning of the myelin sheath, a decrease in the number of myelinated fibers, and the presence of demyelinated axons. Demyelination is often accompanied by axonal degeneration, where the loss of myelin further destabilizes axons, leading to their breakdown. This exacerbates the loss of motor function in the SOD1-G93A mice [155].

In TDP-43 mouse models, myelinating Schwann cells, like motor neurons, exhibit a mislocalization of TDP-43 from the nucleus to the cytoplasm. This mislocalization is associated with impaired cellular functions, which synergistically contributes to the disease process [156]. As well as this, Schwann cells with TDP-43 mislocalization also show signs of disrupted myelination [69]. This includes abnormalities in the formation and maintenance of the myelin sheath, which is critical for proper nerve function [157]. The toxic effects of cytoplasmic TDP-43 aggregates can impair the ability of Schwann cells to effectively myelinate axons. TDP-43 mouse models often exhibit signs of peripheral neuropathy, characterized by demyelination and axonal degeneration in peripheral nerves [158,159]. This peripheral nervous system involvement mirrors the symptoms seen in ALS patients, where muscle weakness and atrophy are linked to peripheral nerve dysfunction.

#### 2.2.2. Non-Myelinating Remak Schwann Cells and Amyotrophic Lateral Sclerosis

There is evidence that Remak Schwann cells directly support motor neurons and can influence axonal transport and survival [160,161,162,163]. Recent studies have shown the association of *Pmp2*^+^ Remak Schwann cells with ChAT^+^ motor neuron axons and that this specific subtype of Remak bundles is significantly reduced in the SOD1G93A model [164]. In ALS, research has shown that these bundles can become disorganized, with axons either being improperly ensheathed or completely lost from their Schwann cell support. This disorganization has been observed in both animal models and post-mortem human ALS tissue. This is particularly relevant for sensory and autonomic neurons, where Remak Schwann cells play a central role. In the SOD1-G93A mouse model, evidence of sensory neuron involvement, such as reduced nerve conduction velocities in unmyelinated fibers, points to non-myelinating Schwann cell dysfunction. CD44 is expressed by Remak Schwann cells in proximal peripheral nerves, and its expression is elevated in response to NMJ plasticity, due to a continual cycle of denervation and reinnervation. Additionally, CD44 colocalizes with neuregulin receptors ErbB2 and ErbB3 and ALS-associated neurodegeneration increases the interaction between CD44 and ErbB3, suggestive of their involvement in perisynaptic Schwann cell plasticity [165]. As well as disorganization, the loss of small axons within Remak bundles has been observed in response to ALS-symptom onset and progression, suggesting that Remak Schwann cell dysfunction may precede or accompany motor neuron degeneration, contributing to the overall neurodegenerative process.

#### 2.2.3. Non-Myelinating Perisynaptic Schwann Cells and Amyotrophic Lateral Sclerosis

Emerging evidence suggests that perisynaptic Schwann cells play an important role in the pathology of ALS. The loss of motor neuron innervation due to disease or neuronal injury reignites the regenerative plasticity program within surviving resistant motor neurons, which undergo a sprouting of nerve terminals to reinnervate previously denervated motor end plates located at NMJs [126]. The process of axonal sprouting is mediated by perisynaptic Schwann cells, which are tightly linked with and located at NMJs [21,26,166]. The occurrence of motor axon denervation is accompanied by the extension of processes from perisynaptic Schwann cells, specifically from denervated endplates to neighboring innervated NMJs. This extension enables them to create temporary scaffolds for guiding terminal sprouts from intact axons to the denervated endplates [166,167]. The extent and degree of functional compensation due to compensatory reinnervation is quite extraordinary, as each motoneuron can innervate and maintain motor units up to four times its normal size, being able to compensate for more than 80% of denervated NMJs [168,169].

Multiple studies have reported morphological and functional abnormalities within perisynaptic Schwann cells in the SOD1-G93A mouse model. While in some defined muscle groups such as the *diaphragm*, neck, EDL, and *soleus*, no alterations in perisynaptic Schwann cell numbers or morphology were observed in end-stage SOD1-G93A mice [170]. However, some studies have shown that just before the onset of denervation in *Gastrocnemius* (GC) and *soleus* (S) muscles, a fraction of NMJs were devoid of perisynaptic Schwann cell bodies and they were enveloped by processes extending from pre-terminal Schwann cells [171]. These NMJs have perisynaptic Schwann cells, with abnormally located cell soma outside of the edge of AChR labeling [171]. With disease progression, denervated NMJs exhibited a complete loss of S100 and P75^NTR^ labeling, indicative of a loss of perisynaptic Schwann cells [36,171,172]. The absence of perisynaptic Schwann cell soma around NMJs in the GC muscle has also been established in yet another SOD1 model, the SOD1-G85R mice [171], and after induced denervation in GC, S, and *plantaris* in asymptomatic SOD1-G93A and SOD1-G85R mice [173,174]. The defined loss of perisynaptic Schwann cells from type IIb muscle fibers, largely innervated by highly vulnerable fast-fatigable motor neurons, has been attributed to the overexpression of Sema3A in perisynaptic Schwann cells. Sema3A is a neuronal regeneration inhibitor, making the neurons at these NMJs vulnerable to phagocytosis by infiltrating macrophages [174,175]. Additionally, electrophysiological measurements of perisynaptic Schwann cells in S (disease resilient) and *sternomastoid* (STM) muscle (disease vulnerable, largely innervated by fast-fatigable motor axons), in pre-symptomatic SOD1-G37R mice revealed that perisynaptic Schwann cells exhibited increased mAChR-dependent activity within STM NMJs [150]. Notably, perisynaptic Schwann cells from both muscles extended unorganized processes from disconnected NMJs and failed to initiate nerve terminal sprouts at disease-vulnerable NMJs, a phenomenon essential for compensatory reinnervation. Additionally, a higher galectin-3 (MAC-2) expression was absent within perisynaptic Schwann cells upon NMJ denervation in an ALS rodent model indicative of defective axonal debris phagocytosis [149,150].

Perisynaptic Schwann cells also exhibit remarkable intracellular changes due to altered motor neuron activity and loss. Notably, perisynaptic Schwann cells display increased intracellular calcium transients and initiate calcium-signaling pathways in response to the neuronal activity-induced release of neurotransmitters [176]. Thus, the ability of terminal Schwann cells to monitor or decipher the extent of neurotransmission at individual synapses enables them to substantially modulate synaptic function and integrity, and consequently the impairment in neuronal transmission frequently observed in ALS also directly impacts NMJ integrity [177].

Furthermore, an underexplored aspect of ALS research is the role of perisynaptic Schwann cells in the agrin/MuSK signaling pathway, which is a crucial determinant in modulating neuromuscular junction (NMJ) stability. Agrin, along with its receptor muscle-specific kinase (MuSK), plays a key role in NMJ assembly and maturation during development (reviewed by Darabid et al., 2014) [178]. Perisynaptic Schwann cells, together with neurons and muscle cells, are known to release agrin, especially in response to denervation [179,180]. Given that increased MuSK activity delays muscle denervation, improves muscle function, and postpones disease onset in SOD1G93A ALS mouse models [181], it is plausible that perisynaptic Schwann cell-mediated agrin synthesis may be compromised in ALS. Moreover, agrin levels are partially regulated by matrix metalloproteinases (MMPs) [178], and perisynaptic Schwann cells may influence these levels through their secretion of MMPs. Importantly, perisynaptic Schwann cells express MMP-3 at the NMJ, with its expression closely linked to the state of innervation, and enhanced agrin expression, due to the genetic deletion of MMP-3, preserves endplates after nerve injury and degeneration [182,183]. Similarly, diminishing neuronal MMP-9 levels via an shRNA-mediated knockdown of MMP significantly delayed muscle denervation [184], highlighting the ability of perisynaptic Schwann cells in regulating NMJ stability levels via agrin and MMP secretion. Negro et al., 2017 reported that that CXCL12α (SDF-1) is generated by perisynaptic Schwann cells after motor axon terminal degeneration, which acts via the neuronal CXCR4 receptor; thus, CXCL12α promotes recovery. Of note, recombinant CXCL12α accelerated neurotransmission recovery and stimulated spinal motor neuron axon growth in vitro, suggestive of the potential of perisynaptic Schwann cells to participate in the recovery from motor axon damage [28]. As hydrogen peroxide (H_2_O_2_) serves as a key pro-regenerative signal in perisynaptic Schwann cells (PSCs), the analyses of H_2_O_2_-induced Schwann cell genes revealed enrichment in extracellular matrix transcripts, including *Connective Tissue Growth Factor* (*Ctgf*) and the inhibition of H_2_O_2_ or *Ctgf*-impaired Schwann cell migration and axon regrowth, delaying neuromuscular recovery. These findings highlight perisynaptic Schwan cell-induced *Ctgf* as crucial pro-regenerative factors during nerve regeneration [185]. While these studies implicate a peripheral importance of Ctgf expression, studies in SOD1-G93A mice have revealed increased levels of CTGF/CCN2 in the skeletal muscle and spinal cord and its inhibition reduced fibrosis in the skeletal muscle of SOD1-G93A mice, concomitantly improving muscle and locomotor performance [186]. Overall, further research is required to elucidate fundamental nerve regeneration and maintenance responses initiated via cross talk between perisynaptic Schwann cells and other cells at the NMJ in physiological and pathological states.

#### 2.2.4. Satellite Glial Cells and Amyotrophic Lateral Sclerosis

Satellite glial cells are predominantly located in the PNS, specifically around the cell bodies of sensory neurons in dorsal root ganglia (DRG) and autonomic ganglia. These cells provide support and protection for neurons, regulate the microenvironment, and are involved in modulating the neuronal response to injury and inflammation. They are also involved in maintaining homeostasis and responding to peripheral nerve damage [30]. Satellite glia play a complex and multifaceted role in the progression of Amyotrophic Lateral Sclerosis (ALS), particularly through their interactions with peripheral neurons and their involvement in neuroinflammatory and neurodegenerative processes [187]. In response to neuronal injury or stress, satellite glia become activated. This activation leads to a reactive state where they can produce and release various pro-inflammatory cytokines, chemokines, and reactive oxygen species (ROS) [188]. In ALS, the chronic activation of satellite glia contributes to a sustained inflammatory environment around peripheral neurons. This inflammatory state exacerbates neuronal damage by promoting oxidative stress, which can harm both neuronal and glial cells [187]. This chronic neuroinflammation mirrors similar neurotransmitter dysregulation throughout the PNS [175]. Satellite glia play a crucial role in maintaining the balance of neurotransmitters around neurons, and their dysfunction can lead to significant dysregulation, contributing to disease progression [188,189]. An important function of satellite glia is in the regulation of neurotransmitter levels by taking up excess neurotransmitters from the synaptic cleft, particularly glutamate, a key excitatory neurotransmitter. In parallel, they are involved in maintaining the balance of ions, such as potassium (K+) and calcium (Ca2+), which are critical for normal neurotransmission and neuronal excitability [30,189]. In ALS, satellite glia become dysfunctional, leading to neurotransmitter imbalances, particularly with glutamate, contributing to motor neuron degeneration. Moreover, satellite glia may lose their ability to properly buffer and uptake excess glutamate, leading to elevated levels of extracellular glutamate around motor neurons more distally in the PNS. High levels of glutamate overstimulate glutamate receptors (such as NMDA and AMPA receptors) on neurons, leading to excessive calcium influx. This triggers a cascade of events that ultimately cause neuronal damage and death, via excitotoxicity [150]. Notably, motor neurons are particularly susceptible to excitotoxicity because of their high metabolic demands and relatively low capacity for handling prolonged excitatory stimulation. The failure of satellite glia to regulate glutamate levels can thus significantly accelerate motor neuron degeneration in ALS.

Furthermore, satellite glia are also involved in buffering extracellular potassium levels, which fluctuate during neuronal activity [190]. In ALS, impaired potassium regulation by satellite glia could eventually lead to abnormal neuronal excitability, further contributing to motor neuron dysfunction. Dysregulated calcium signaling, partly influenced by dysfunctional satellite glia, is another key factor in neurotransmitter dysregulation. It is well established that abnormal calcium handling exacerbates excitotoxicity and contributes to neuronal degeneration. In ALS, impaired calcium handling within motor neurons and muscles is strongly compromised, leading to impaired neurotransmission [191,192]. Additionally, satellite glia communicate with neurons and with each other through gap junctions, which are channels that allow ions and small molecules to pass directly between cells. This communication helps in the buffering of neurotransmitters and maintaining the ionic environment [193]. In ALS, gap junctions between satellite glia may become dysfunctional, leading to impaired intercellular communication and disrupted neurotransmitter regulation. This can worsen neurotransmitter imbalances and contribute to the progression of neurodegeneration.

While rodent models have enabled us to shed insights into the precise contribution of Schwann cell and their dysfunction to ALS, most of these models only recapitulate to a limited extent the human fALS pathology. Moreover, the majority of ALS pathology remains sporadic with an unknown genetic etiology, thus making it important to assess the role of Schwann cells in sporadic ALS. In a sporadic ALS patient, postmortem analyses revealed p-TDP-43-immunoreactive inclusions localized in the cytoplasm of Schwann cells [194], indicative of a conserved Schwann cell impairment in sporadic ALS. Further careful assessments of postmortem tissues and sporadic ALS patient-derived model systems will enable a better understanding of Schwann cell pathology in ALS.

## 3. New Perspectives for Amyotrophic Lateral Sclerosis: Targeting Schwann Cells for Therapeutic Delivery

### 3.1. Targeting Schwann Cells for Therapeutic Delivery: Lessons Learned from CMT1

Therapies targeting Schwann cells and particularly myelinating Schwann cells have been a hot topic for CMT1 as the field moves into clinical trials for this disease (see several excellent reviews [195,196,197,198,199]). In brief, multiple approaches have been taken to develop candidate therapeutics to treat CMT1 including small molecule drugs and gene therapies. Small molecule drugs for CMT1 generally have not been designed to specifically target Schwann cells given their oral administration but instead require an efficient crossing of the blood–nerve barrier [195,200]. New and recent small molecule clinical trials for human CMT1 patients include NMD670 to improve NMJ function (ongoing [201], ClinicalTrials.gov ID NCT06482437), PXT3003 to reduce PMP22 expression in CMT1A (ongoing [202], ClinicalTrials.gov ID NCT03023540) and ACE-083 to improve muscle mass (terminated due to not achieving the predefined endpoints [203], ClinicalTrials.gov ID NCT03124459). Additionally, large molecule drugs, including gene therapies, are gaining popularity as treatments for CMT1 but ideally need to be targeted to the appropriate tissues and cells. Multiple approaches have been employed and evaluated in CMT1 rodent models including the intrathecal, intravenous, or intra-nerve injection of candidate viral (Lentiviral or Adeno-associated viral (AAV)) or non-viral gene therapies. Preclinical viral therapies are in development for CMT1A (shRNA, miRNA, or siRNA delivery), CMT1B (transgene delivery) and CMT1X (transgene delivery) and although both lentiviral- and AAV-based therapies have been created, AAV is considered a more translatable approach given that it exhibits persistent and superior transgene expression [198]. Several AAV serotypes have been tested for their ability to transduce myelinating Schwann cells and satellite glial cells with variable effects depending on the species tested but AAV9 has emerged as the preferred serotype for CMT1 [197,198]. Depending on the therapy being delivered, there may or may not be a necessity for Schwann cell-specific targeting which can be addressed by using a Schwann cell-specific promoter (i.e., *MPZ* promoter for myelinating Schwann cells) [198]. Although several gene therapies are close to entering clinical trials in humans, the only viral gene therapy trial to date for human CMT1 patients is scAAV1.tMCK.NTF3 for the treatment of CMT1A (ClinicalTrials.gov ID NCT03520751) which is delayed due to challenges with vector production [204]. Non-viral gene therapies are also being developed for CMT1A including a subcutaneous injection of ASOs, an intravenous injection of siRNA encapsulated by squalenoyl nanoparticles, and an intra-nerve injection of CRISPR/Cas9 targeting the super enhancer in the *PMP22* promoter which all remain under preclinical evaluation [198]. As with viral gene therapies, there is much interest with developing non-viral delivery vectors that efficiently target myelinating Schwann cells [205]. Squalenoyl nanoparticles and fatty acids conjugated onto siRNAs are two vectors that are currently being developed as CMT1A therapies [198,206,207]. Cellular therapies are also being considered for CMT1 with multiple clinical trials ongoing (EN001: ClinicalTrials.gov ID NCT06328712 and ClinicalTrials.gov ID NCT06218134 and CLZ-2002: ClinicalTrials.gov ID NCT05947578).

While significant progress has been made in developing candidate therapies for CMT1 and targeting them to myelinating Schwann cells, further research is crucial to overcome the current challenges, including selective delivery, blood–nerve barrier penetration, peripheral nervous system distribution, and long-term efficacy and safety, to develop effective treatments for demyelinating diseases. Additionally, the therapeutic delivery for CMT1 has primarily focused on myelinating Schwann cells. As we learn more about the pathophysiology of this disease, other types of Schwann cells may also become critical targets for therapeutic delivery. However, viral and non-viral vectors targeting satellite glial cells and especially Remak Schwann cells and terminal Schwann cells remains a gap in our knowledge.

### 3.2. Targeting Schwann Cells for Therapeutic Delivery in Amyotrophic Lateral Sclerosis

Pharmacological and gene therapy approaches targeting Schwann cells and their associated signaling pathways (e.g., c-Kit, CSF-1R, and iNOS) are attractive and viable options for developing novel ALS therapies, thus potentially addressing peripheral nerve inflammation and pathology more effectively. Gene therapy targeting multiple types of Schwann cells holds promise for ALS by addressing peripheral nerve inflammation, supporting nerve repair, and potentially modifying disease progression. A subset of proliferative Schwann cells (Ki67+) in the sciatic nerves of the SOD1G93A rat model of ALS was found to express c-Kit, suggesting that this receptor may drive Schwann cell proliferation in ALS. Interestingly, c-Kit+ mast cells were also identified in human ALS patient sciatic nerves but were absent in healthy controls, implicating an ALS-specific inflammatory pathway involving c-Kit signaling [208]. Masitinib treatment in SOD1G93A rats led to the inhibition of CSF-1R and c-Kit, reduced Schwann cell reactivity, and immune cell infiltration in both sciatic nerves and ventral roots. This reduction points to a mechanism by which masitinib may alleviate peripheral nerve pathology, thus making it a promising candidate for an ALS therapy [208]. An initial Phase 2/3 clinical trial, AB10015 (ClinicalTrials.gov ID NCT02588677), enrolled 394 people with ALS whose symptoms had begun in the previous three years, and patients received either placebo or masitinib for 48 weeks up to one year. The daily dose of masitinib administered was 3 or 4.5 mg/kg, and it was given as an add-on to Rilutek (Riluzole). Recent results of the trial indicate a 27% reduction in disease progression. Notably, the maximum benefit of a 42% slowed rate of disease progression was observed in normal progressors displaying mild or moderate ALS, making masitinib a promising drug therapy [209,210]. Further, modifying the c-Kit pathway through gene therapy could reduce maladaptive proliferation and associated inflammation. Using gene-editing tools, such as CRISPR/Cas9 or RNA interference (RNAi), to downregulate c-Kit expression specifically in Schwann cells may reduce pathological cell proliferation and inflammatory signaling. Given the role of CSF-1R in immune cell recruitment to ALS-affected nerves, as well as CSF-1R inhibitors, gene therapy could be designed to downregulate or inhibit CSF-1R expression in Schwann cells, limiting immune cell infiltration and thus reducing neuroinflammation in peripheral nerves [208,211]. 

Expression of inducible nitric oxide synthase (iNOS) is increased in Schwann cells of human ALS peripheral nerves, particularly at the paranodal regions of Nodes of Ranvier [212]. Treatment of SOD1-G93A mice with drugs selectively inhibiting iNOS delayed disease onset and extended survival in SOD1-G93A mice, further supporting the role of SC-driven inflammation in ALS pathology [212]. Using RNAi or antisense oligonucleotides (ASOs) to selectively knockdown iNOS in Schwann cells could decrease oxidative stress and improve neuronal survival [213]. Gene therapies that target iNOS could help preserve the integrity of the nodes of Ranvier, which are critical for nerve conduction. A crucial therapeutic direction to focus on would be towards facilitating axonal repair and regeneration via gene therapy-mediated enhancement of remyelination. This could be achieved by selectively modulating pathways that promote Schwann cell differentiation and myelination to support axonal health. For example, c-Jun levels in myelinating Schwann cells decline with age and exhibit a reduced capacity for regeneration upon nerve injury, making c-Jun an attractive target for promoting the regeneration of myelinating Schwann cells [214]. Additionally, myelinating Schwann cells contribute to the extracellular matrix around peripheral nerves, which is crucial for nerve repair, thus modulating the extracellular matrix components could be of benefit [215]. Gene therapies that upregulate regenerative components of the extracellular matrix, such as laminins or fibronectins, which are reduced in ALS, may facilitate nerve repair and functional recovery in ALS [215,216,217]. In conclusion, a strong emphasis on research and clinical trials is needed to optimize these strategies and ensure their safety and efficacy in patients with ALS.

## 4. Conclusions

Schwann cells are emerging as integral contributors to the progression of ALS, influencing neuroinflammation, demyelination, and axonal degeneration. Their dysfunction contributes not only to motor neuron degeneration but also to compromised nerve repair and synaptic plasticity. Current therapeutic strategies being developed to target Schwann cells for CMT1 hold promise for ALS treatment by potentially enhancing remyelination, regulating inflammation, and supporting axonal regeneration. Future research should prioritize refining these approaches to optimize their therapeutic efficacy and safety and advancing our understanding of Schwann cell pathology to support the development of effective interventions against ALS. The parallels between ALS and CMT are also worth highlighting, as both fields have the potential to yield valuable insights that could mutually advance understanding and treatment approaches.

## Figures and Tables

**Table 1:** CMT1 is caused by the mutation of genes affecting myelinating Schwann cells. The gene, gene function, CMT1 subtype, mutation type/location, and patient symptom severity are listed for each known CMT1 gene.

Gene	Gene Function	CMT1 Subtype	Mutation Type & Location	CMT Severity
*Peripheral myelin protein 22* (*PMP22*)	Myelin architecture regulation (undefined role in cell adhesion), Schwann cell proliferation and survival	CMT1A	Gene Duplication	Classic CMT
		HNPP	Gene Deletion	Mild CMT
		CMT1E	Point mutations and small insertions/deletions, primarily localized to transmembrane domains, some in extracellular domains	Severe, Classic or Mild CMT depending on the mutation
*Myelin protein zero* (MPZ/P0)	Adhesion protein required for compaction of myelin lamellae	CMT1B	Numerous point mutations identified, primarily localized to the extracellular domain	Severe or Classic CMT
		CMT2I		Adult-onset, potentially rapidly progressive
		CMT2J		Adult-onset with hearing loss and pupillary abnormalities, potentially rapidly progressive
*Lipopolysaccharide-induced tumor* *necrosis factor-* *alpha factor* (*LITAF*/*SIMPLE*)	Recruitment of ESCRT components for endosomal trafficking and signaling	CMT1C	Point mutations, primarily localized to the C-terminal cysteine-rich domain	Classic CMT
*Early growth* *response 2* (*EGR2*/*Krox20*)	Master transcription factor controlling Schwann cell myelination	CMT1D	Point mutations, primarily localized in the zinc finger domains	Severe or Classic CMT
*Neurofilament light polypeptide* (*NEFL*)	Neuronal cytoskeletal protein involved in regulating axon caliber	CMT1F	Point mutations, localized throughout the protein	Severe or Classic CMT, hearing loss
		CMT2E		
*Peripheral myelin protein 2* (*PMP2*)	Myelin sheath stiffness, membrane stacking, and lipid transfer	CMT1G	Point mutations, primarily localized to the fatty acid binding pocket	Classic CMT
*Fibulin 5 gene* (*FBLN5*)	Extracellular matrix protein	CMT1H	Point mutations, few identified to date	Classic or Mild CMT
*Polymerase III, RNA, subunit B* (*POLR3B*)	Subunit of RNA polymerase transcribing non-coding RNA, also involved in RNA processing and translation	CMT1I	Point mutations, few identified to date	Severe CMT, also intellectual disability, spasticity, and ataxia
*Inositol 1,4,5-trisphosphate receptor, type 3* (*ITPR3*)	Receptor for inositol triphosphate (IP3), intracellular calcium release	CMT1J	Point mutations, few identified to date	Severe or Classic CMT
*Gap junction protein beta 1* (*GJB1*/*Cx32*)	Gap junction hemichannel involved in transporting metabolites and signaling molecules throughout myelin	CMT1X	Numerous point mutations identified, localized throughout the protein	Classic CMT, males more affected than females, more frequent hand deformities and CNS symptoms

## Data Availability

No new data were created or analyzed in this study. Data sharing is not applicable to this article.
